# Supplementary material for: Loneliness trajectories over three decades are associated with conspiracist worldviews in midlife
Source: Nat Commun. 2024 Apr 29;15:3629. doi: 10.1038/s41467-024-47113-x (PMC11059163; doi:10.1038/s41467-024-47113-x)
Supplement: Supplementary file 1 — Supplementary Information [file 41467_2024_47113_MOESM1_ESM.pdf]

Supplementary Information for

Loneliness trajectories over three decades predict conspiracist worldviews in midlife

Kinga Bierwiaczonek[1]*, Anne-Marie Fluit[1], Tilmann von Soest[1,2], Matthew J. Hornsey[3], Jonas R. Kunst[1].

Correspondence to: k.m.bierwiaczonek@psykologi.uio.no

Supplementary Table 1.
Items of study variables

Measure	Items	Reliability (α)				
		T1	T2	T3	T4	T5
Loneliness (UCLA Loneliness Scale, short version, Russel et al., 1980); 4-point response scale		.78	.80	.76	.78	.80
	1. No one really knows me well*					
	2. People are around me but not with me*					
	3. I feel lonely**					
Conspiracy worldview (Conspiracy Mentality Questionnaire, Bruder et al., 2013); 7-point response scale		-	-	-	-	.83
	1. Many very important things happen in the world, which the public is never informed about.					
	2. Politicians usually do not tell us the true motives for their decisions.					
	3. Government agencies closely monitor all citizens.					
	4. Events which superficially seem to lack a connection are often the result of secret activities.					
	5. There are secret organizations that greatly influence political decisions.					
Parental education (assessed for mother and father; listed are response options)		-	-	-	-	-
	1. 7-years primary/secondary school (or less)					
	2. Junior high school/comprehensive school					
	3. High school/upper secondary school (general course)					
	4. High school/upper secondary school (vocational course)					
	5. Vocational training in craft, industry, farming etc.					
	6. University or other lengthy education					
	7. University or other lengthy education					
	8. Other education					
	9. Unsure/don't know					
Political orientation (items from Materialism–Post-Materialism measure; Inglehart, 1977); 10-point response scale		-	.63	-	-	-
	1. Maintaining a high rate of economic growth					
	2. Make sure this country has strong defense forces					
	3. Stop mixing of races					
	4. Make Norway available to receive more refugees and immigrants					
Anxiety and depression symptoms (Hopkins Symptoms Checklist; Derogatis, 1982; Derogatis et al., 1974) ; 4-point response scale		.87	.88	.83	.90	.91
	1. Suddenly scared for no reason					
	2. Constantly scared or worried					
	3. Faintness or dizziness					
	4. Nervousness or shakiness inside					
	5. Crying easily					
	6. Blaming yourself for things					
	7. Felt to tired to do things					
	8. Had trouble sleeping					
	9. Felt unhappy, sad, or depressed					
	1. Felt hopeless about the future					
	11. Felt tense or keyed up					
	12. Worried too much about things					

Note. * These two items were previously shown to form an emotional loneliness subdimension in the Norwegian short version of the UCLA loneliness scale[37]; ** This item is a direct measure of loneliness and is part of the Norwegian short version of the scale[37].

Supplementary Table 2.

Descriptive statistics, normality tests and correlations between study variables

		Mean	SD	1	2	3	4	5	6
1	Loneliness T1	2.070	.718						
2	Loneliness T2	2.083	.736	.564, $p < .001$					
3	Loneliness T3	2.186	.658	.377, $p < .001$	.474, $p < .001$				
4	Loneliness T4	2.137	.658	.328, $p < .001$	.392, $p < .001$	.498, $p < .001$			
5	Loneliness T5	2.198	.691	.284, $p < .001$	.325, $p < .001$	.426, $p < .001$	.524, $p < .001$		
6	Depression/Anxiety Symptoms T1	1.599	.459	.434, $p < .001$	.314, $p < .001$	.265, $p < .001$	.215, $p < .001$	.218, $p < .001$	
7	Depression/Anxiety Symptoms T2	1.620	.486	.334, $p < .001$	.385, $p < .001$	.309, $p < .001$	.235, $p < .001$	.237, $p < .001$	.592, $p < .001$
8	Depression/Anxiety Symptoms T3	1.568	.489	.243, $p < .001$	.248, $p < .001$	.465, $p < .001$	.337, $p < .001$	.271, $p < .001$	.394, $p < .001$
9	Depression/Anxiety Symptoms T4	1.473	.463	.214, $p < .001$	.220, $p < .001$	.306, $p < .001$	.496, $p < .001$	.386, $p < .001$	.305, $p < .001$
10	Depression/Anxiety Symptoms T5	1.612	.511	.207, $p < .001$	.182, $p < .001$	.257, $p < .001$	.357, $p < .001$	.557, $p < .001$	.290, $p < .001$
11	Age at T5 (registry data)	43.220	2.000	.103, $p < .001$	.030, $p = .165$	-.040, $p = .072$	-.046, $p = .031$	-.024, $p = .259$	.095, $p < .001$
12	Sex (registry data)	-	-	.100, $p < .001$	.078, $p < .001$	.091, $p < .001$	.073, $p < .001$	.078, $p < .001$	.244, $p < .001$
13	Parental Education T1	2.438	1.028	-.025, $p = .287$	.003, $p = .915$	-.055, $p = .025$	-.022, $p = .344$	-.041, $p = .081$	-.017, $p = .484$
14	Political Orientation T2	5.408	1.707	-.134, $p < .001$	-.117, $p < .001$	-.074, $p = .002$	-.081, $p < .001$	-.034, $p = .138$	-.142, $p < .001$
15	Conspiracy Worldview T5	3.776	1.310	.003, $p = .888$	.052, $p = .019$	.060, $p = .008$	.074, $p < .001$	.126, $p < .001$	.052, $p = .020$

Note. All *p*-values are two-tailed.

Supplementary Table 2 (continued)

		7	8	9	10	11	12	13	14
1	Loneliness T1								
2	Loneliness T2								
3	Loneliness T3								
4	Loneliness T4								
5	Loneliness T5								
6	Depression/Anxiety Symptoms T1								
7	Depression/Anxiety Symptoms T2								
8	Depression/Anxiety Symptoms T3	.461, *p* < .001							
9	Depression/Anxiety Symptoms T4	.369, *p* < .001	.490, *p* < .001						
10	Depression/Anxiety Symptoms T5	.343, *p* < .001	.404, *p* < .001	.534, *p* < .001					
11	Age at T5 (registry data)	.050, *p* = .022	-.089, *p* < .001	-.043, *p* = .046	-.045, *p* = .037				
12	Sex (registry data)	.325, *p* < .001	.227, *p* < .001	.140, *p* < .001	.177, *p* < .001	.032, *p* = .132			
13	Parental Education T1	-.036, *p* = .128	-.032, *p* = .192	-.003, *p* = .900	-.032, *p* = .167	.014, *p* = .540	-.002, *p* = .946		
14	Political Orientation T2	-.143, *p* < .001	-.078, *p* = .001	-.057, *p* = .013	-.080, *p* < .001	-.095, *p* < .001	-.207, *p* < .001	-.190, *p* < .001	
15	Conspiracy Worldview T5	.099, *p* < .001	.075, *p* < .001	.083, *p* < .001	.118, *p* < .001	-.050, *p* = .021	.033, *p* = .126	-.172, *p* < .001	.226, *p* < .001

Note. All *p*-values are two-tailed.

Supplementary Table 3.

Measurement invariance tests of the three-item loneliness measure.

Model	χ^2	*df*	$p(\chi^2)$	CFI	TLI	SRMR	RMSEA	90% CI_{RMSEA}		p_{close}
								lower	upper	
Configural invariance	76.525	50	<.001	.998	.996	.016	.015	.008	.022	1.000
Weak invariance	92.304	58	.003	.997	.995	.020	.016	.010	.022	1.000
Strong invariance	29.363	70	<.001	.982	.973	.031	.038	.033	.042	1.000

Note. The configural invariance model includes all observed indicators loading freely on the latent loneliness construct at each time point. In the weak invariance model, factor loadings are constrained to equality across time points. In the strong invariance model, required for latent growth modeling, loadings and intercepts of the items are constrained to equality across time points. The excellent fit with the data of the strong invariance model indicates that the construct of loneliness remained invariant between 1992 and 2020. χ^2 – chi-square, *df* – degrees of freedom; CFI – comparative fit index; TLI – Tucker-Lewis index; SRMR – standardized root mean squared residual; RMSEA – root mean square error of approximation; 90% CI_{RMSEA} – 90% confidence interval around RMSEA; p_{close} – *p*-value of close fit testing the null hypothesis that RMSEA = .05 (i.e., that the model is close-fitting).

Supplementary Table 4.

Model 1 and 2: Results of second-order latent growth curve analyses, unconditional linear and quadratic trajectories

Model Estimates	Variable(s)	Model 1: Linear Change					Model 2: Quadratic Change				
		Estimate	*SE*	*p*	95% CI_{lower}	95% CI_{upper}	Estimate	*SE*	*p*	95% CI_{lower}	95% CI_{upper}
Means											
	Linear Slope	.036	.006	<.001	.026	.045	.058	.021	.006	.023	.093
	Quadratic Slope						-.007	.007	.258	-.018	.003
Variances											
	Intercept	.231	.012	<.001	.211	.251	.301	.017	<.001	.237	.328
	Linear Slope	.042	.004	<.001	.036	.047	.316	.037	<.001	.255	.377
	Quadratic Slope						.024	.005	<.001	.016	.033
Correlations											
	Intercept with Linear Slope	-.375	.030	<.001	-.424	-.326	-.566	.033	<.001	-.620	-.511
	Intercept with Quadratic Slope						.486	.054	<.001	.397	.576
	Linear Slope with Quadratic Slope						-.937	.033	<.001	-.992	-.883
Model Fit											
		χ^2 (76) = 358.185, *p* < .001					χ^2 (72) = 281.952, *p* < .001; $\Delta\chi^2$(4) = 76.233, *p* < .001				
		CFI = .977					CFI = .983				
		TLI = .969					TLI = .975				
		RMSEA = .041, 90% CI_{RMSEA} = [.037, 045], p_{close} = 1.000					RMSEA = .036, 90% CI_{RMSEA} = [.032, 041], p_{close} = 1.000				
		SRMR = .041					SRMR = .033				

Note. Models 1 and 2 refer to univariate models including only loneliness, tested to determine the shape of its trajectory. The quadratic change model (Model 2) showed a better fit than the linear change model (Model 1) and was therefore retained. Model 2 indicated that in the overall sample, loneliness tended to increase from adolescence toward adulthood (as indicated by the significant, positive mean of the linear slope), and this increase was relatively steady (non-significant mean of the quadratic slope). Intercept – estimated initial level of loneliness; linear slope – linear change (here, increase, as indicated by positive values) of loneliness; quadratic slope – the acceleration or deceleration of the change expressed by linear slope (i.e., the extent to which the decrease of loneliness slows down or accelerates over time); χ^2 – chi-square, *df* – degrees of freedom; CFI – comparative fit index; TLI – Tucker-Lewis index; SRMR – standardized root mean squared residual; RMSEA – root mean square error of approximation; 90% CI_{RMSEA} – 90% confidence interval around RMSEA; p_{close} – *p*-value of close fit testing the null hypothesis that RMSEA = .05 (i.e., that the model is close-fitting). For means and variances, unstandardized estimates are presented, whereas for correlations and regressions, standardized estimates are presented. All *p*-values are two-tailed and based on the critical value of the *z*-score obtained by dividing the estimate by its standard error. Since the reported analysis is a latent growth curve model with one outcome, adjustments for multiple comparisons are not applicable.

Supplementary Table 5.

Model 3: Results of second-order latent growth curve analyses controlling for time-invariant covariates

Model Estimates	Variable(s)	Estimate	SE	*p*	95% CI_{lower}	95% CI_{upper}
Means						
	Linear Slope	0.061	0.021	0.004	0.026	0.096
	Quadratic Slope	-0.008	0.007	0.208	-0.019	0.003
Variances						
	Intercept	0.303	0.017	<.001	0.275	0.331
	Linear Slope	0.304	0.038	<.001	0.242	0.367
	Quadratic Slope	0.02	0.005	<.001	0.012	0.028
Correlations						
	Intercept with Linear Slope	-0.574	0.034	<.001	-0.63	-0.519
	Intercept with Quadratic Slope	0.529	0.062	<.001	0.427	0.632
	Linear Slope with Quadratic Slope	-0.966	0.041	<.001	-1.034	-0.898
Regressions						
	Intercept --> Conspiracy Mentality T5	0.135	0.033	<.001	0.081	0.189
	Linear Slope --> Conspiracy Mentality T5	0.149	0.041	<.001	0.081	0.217
	Age --> Conspiracy Mentality T5	-0.008	0.024	0.729	-0.048	0.031
	Sex (Female) --> Conspiracy Mentality T5	0.093	0.023	<.001	0.055	0.131
	Parental Education at T1 --> Conspiracy Mentality T5	-0.146	0.025	<.001	-0.186	-0.105
	Political Orientation at T2 --> Conspiracy Mentality T5	0.251	0.025	<.001	0.21	0.292
Model Fit						
		χ^2 (214) = 1,047.096, p < .001				
		CFI = .952				
		TLI = .939				
		RMSEA = .042, 90% CI_{RMSEA} = [.039, 044], p_{close} = 1.000				
		SRMR = .034				

Note. With Model 3, we tested the robustness of results to time-invariant covariates (sex and age at T5 as recorded in national registries, political orientation at T2, parental education at T1). After including covariates, the slope and intercept of loneliness were still significantly related to conspiracy worldviews in 2020, demonstrating the robustness of the results. Intercept – estimated initial level of loneliness; linear slope – linear change (here, increase, as indicated by positive values) of loneliness; quadratic slope – the acceleration or deceleration of the change expressed by linear slope (i.e., the extent to which the decrease of loneliness slows down or accelerates over time); χ^2 – chi-square, *df* – degrees of freedom; CFI – comparative fit index; TLI – Tucker-Lewis index; SRMR – standardized root mean squared residual; RMSEA – root mean square error of approximation; 90% CI_{RMSEA} – 90% confidence interval around RMSEA; p_{close} – *p*-value of close fit testing the null hypothesis that RMSEA = .05 (i.e., that the model is close-fitting). For means and variances, unstandardized estimates are presented, whereas for correlations and regressions, standardized estimates are presented. All *p*-values are two-tailed and based on the critical value of the *z*-score obtained by dividing the estimate by its standard error. Since the reported analysis is a latent growth curve model with one outcome, adjustments for multiple comparisons are not applicable.

Supplementary Table 6.

Model 4: Results of second-order latent growth curve analyses controlling for depression and anxiety symptoms as time-variant covariates

Model Estimates	Variable(s)	Estimate	*SE*	*p*	95% CI_{lower}	95% CI_{upper}
Means						
	Linear Slope	-.020	.178	.910	-.313	0.272
	Quadratic Slope	.008	.111	.945	-0.175	.191
Variances						
	Intercept	.261	.015	<.001	.236	.287
	Linear Slope	.283	.032	<.001	.229	.336
	Quadratic Slope	.016	.004	<.001	.009	.023
Correlations						
	Intercept with Linear Slope	-.605	.037	<.001	-.666	-.545
	Intercept with Quadratic Slope	.598	.083	<.001	.461	.735
	Linear Slope with Quadratic Slope	-1.025	.052	<.001	-1.110	-.939
Regressions						
	Intercept --> Conspiracy Mentality T5	.110	.033	.001	.056	.164
	Linear Slope--> Conspiracy Mentality T5	.110	.045	.015	.035	.185
	Age --> Conspiracy Mentality T5	-.016	.024	.488	-.055	.022
	Sex (Female) --> Conspiracy Mentality T5	.096	.023	<.001	.058	.134
	Parental Education at T1 --> Conspiracy Mentality T5	-.147	.025	<.001	-.188	-.107
	Political Orientation at T2 --> Conspiracy Mentality T5	.248	.025	<.001	.207	.288
	Hopkins Symptom Checklist T1 → Loneliness T1	.217	.031	<.001	.166	.268
	Hopkins Symptom Checklist T2 --> Loneliness T2	.230	.022	<.001	.193	.267
	Hopkins Symptom Checklist T3 --> Loneliness T3	.312	.023	<.001	.275	.350
	Hopkins Symptom Checklist T4 --> Loneliness T4	.299	.025	<.001	.241	.357
	Hopkins Symptom Checklist T5 --> Loneliness T5	.303	.247	.219	-.103	.709
Model Fit						
		χ^2 (324) = 2,134.056, p < .001				
		CFI = .907				
		TLI = .893				
		RMSEA = .050, 90% CI_{RMSEA} = [.048, 052], p_{close} = .424				
		SRMR = .038				

Note. With Model 4, we tested the robustness of results to symptoms of depression and anxiety as time-varying covariate. After including these covariates, the slope and intercept of loneliness were still significantly related to conspiracy worldviews in 2020, demonstrating the robustness of the results. Intercept – estimated initial level of loneliness; linear slope – linear change (here, increase, as indicated by positive values) of loneliness; quadratic slope – the acceleration or deceleration of the change expressed by linear slope (i.e., the extent to which the decrease of loneliness slows down or accelerates over time); χ^2 – chi-square, *df* – degrees of freedom; CFI – comparative fit index; TLI – Tucker-Lewis index; SRMR – standardized root mean squared residual; RMSEA – root mean square error of approximation; 90% CI_{RMSEA} – 90% confidence interval around RMSEA; p_{close} – *p*-value of close fit testing the null hypothesis that RMSEA = .05 (i.e., that the model is close-fitting). For means and variances, unstandardized estimates are presented, whereas for correlations and regressions, standardized estimates are presented. All *p*-values are two-tailed and based on the critical value of the *z*-score obtained by dividing the estimate by its standard error. Since the reported analysis is a latent growth curve model with one outcome, adjustments for multiple comparisons are not applicable.

Supplementary Note 1.
Data are available in the file MData_Invariance.csv available via OSF (https://osf.io/yjzqe). Please note that variable names are not provided in the file because of Mplus requirements. Variable names are:
CMQ5_1 – CMQ5_5: conspiracy worldview at T5 (items);
UCLA1_1 – UCLA5_5: loneliness at T1 – T5 (items);
Con_T2: political orientation at T2;
anx_T1 – dep_T5: anxiety and depression symptoms at T1 – T5.
In line with Norwegian law and regulations, sociodemographic factors used as covariates in some analyses (age, gender, parental education) are not included in the dataset to ensure the full anonymity of participants.
