# Supplementary material for: Loneliness trajectories over three decades are associated with conspiracist worldviews in midlife
Source: Nat Commun. 2024 Apr 29;15:3629. doi: 10.1038/s41467-024-47113-x (PMC11059163; doi:10.1038/s41467-024-47113-x)
Supplement: Supplementary file 3 — Reporting Summary [file 41467_2024_47113_MOESM3_ESM.pdf]

nature portfolio

Corresponding author(s): Kinga Bierwiaczonek

Last updated by author(s): 2024/02/01

Reporting Summary

Nature Portfolio wishes to improve the reproducibility of the work that we publish. This form provides structure for consistency and transparency in reporting. For further information on Nature Portfolio policies, see our Editorial Policies and the Editorial Policy Checklist.

Statistics

For all statistical analyses, confirm that the following items are present in the figure legend, table legend, main text, or Methods section.

n/a	Confirmed	
☐	☒	The exact sample size (*n*) for each experimental group/condition, given as a discrete number and unit of measurement
☐	☒	A statement on whether measurements were taken from distinct samples or whether the same sample was measured repeatedly
☐	☒	The statistical test(s) used AND whether they are one- or two-sided<br>*Only common tests should be described solely by name; describe more complex techniques in the Methods section.*
☐	☒	A description of all covariates tested
☐	☒	A description of any assumptions or corrections, such as tests of normality and adjustment for multiple comparisons
☐	☒	A full description of the statistical parameters including central tendency (e.g. means) or other basic estimates (e.g. regression coefficient) AND variation (e.g. standard deviation) or associated estimates of uncertainty (e.g. confidence intervals)
☐	☒	For null hypothesis testing, the test statistic (e.g. *F*, *t*, *r*) with confidence intervals, effect sizes, degrees of freedom and *P* value noted<br>*Give P values as exact values whenever suitable.*
☒	☐	For Bayesian analysis, information on the choice of priors and Markov chain Monte Carlo settings
☐	☒	For hierarchical and complex designs, identification of the appropriate level for tests and full reporting of outcomes
☒	☐	Estimates of effect sizes (e.g. Cohen's *d*, Pearson's *r*), indicating how they were calculated

Our web collection on statistics for biologists contains articles on many of the points above.

Software and code

Policy information about availability of computer code

Data collection	Data were collected in a paper and pencil format (T1 - T3) and with the Nettskjema data collection tool (T4 - T5), which is the only tool approved at the University of Oslo for collecting sensitive data. More information: https://www.uio.no/english/services/it/adm-services/nettskjiema/about-nettskiema.html
Data analysis	Data were analyzed with Mplus v. 8. Analysis codes are available via the Open Science Framework (https://osf.io/yjzqe)

For manuscripts utilizing custom algorithms or software that are central to the research but not yet described in published literature, software must be made available to editors and reviewers. We strongly encourage code deposition in a community repository (e.g. GitHub). See the Nature Portfolio guidelines for submitting code & software for further information.

Data

Policy information about availability of data

All manuscripts must include a data availability statement. This statement should provide the following information, where applicable:

- Accession codes, unique identifiers, or web links for publicly available datasets
- A description of any restrictions on data availability
- For clinical datasets or third party data, please ensure that the statement adheres to our policy

The primary data generated in this study have been deposited in the Open Science Framework repository under accession code https://osf.io/yjzqe (see

Supplementary Information, Supplementary Note 1, for variable names). The raw data related to sociodemographic characteristics of the participants (age, gender, parental education) are protected and are not available due to Norwegian data privacy laws.

Research involving human participants, their data, or biological material

Policy information about studies with human participants or human data. See also policy information about sex, gender (identity/presentation), and sexual orientation and race, ethnicity and racism.

Reporting on sex and gender

Participant sex in our analyses was taken from the national population registry and refers to biological sex. Sex was used as covariate in additional analyses reported in Supplementary Information (Table S5, S6).

Reporting on race, ethnicity, or other socially relevant groupings

Most participants (93.6%) were ethnic Norwegians; 6.4% of participants had some other socially relevant immigrant background (i.e., were born abroad or had at least one parent who was born abroad). The socially relevant groupings used in this study were parental education and political orientation. Both were included in analyses as time-invariant covariates (see Supplementary Information, Table S5, S6) because previous studies showed that they may be related to belief in conspiracy theories. In addition, we controlled for psychopathology symptoms (anxiety, depression) as time-varying covariates (i.e., we regressed loneliness scores on them within each wave) because previous research linked such symptoms to both conspiracy beliefs and loneliness.

Population characteristics

Participants were junior and senior high school students in grades 7-12 (M = 15.05, SD= 1.98) at the first timepoint (in 1992), and in their early to mid-forties at the last timepoint (in 2020; M = 43.22, SD = 2.00). 57.4% of participants were women, and 42.6% were men. Less than half of participants (43.3%) had at least one parent who attended college or university. These characteristics were controlled for in our analyses.

Recruitment

At T1 (1992), a national sample of Norwegian junior and senior high school students, from 67 schools in grades 7-12 (age 12-20), was selected from stratified areas. Each grade was equally represented, and cluster-sampling was applied with the school as the unit. Most attrition resulted from two factors: (1) four schools included at T1 could not be followed up at T2, and (2) about half of participants changed schools between T1 and T2, and these participants were not followed up further (see the section Behavioural & social sciences study design, Non-participation, for more details). This attrition was unlikely to produce self-selection effects.

Ethics oversight

The study obtained ethical approval from the Regional Committees for Medical Research Ethics (reference no.: 25462; project name: Young in Norway).

Note that full information on the approval of the study protocol must also be provided in the manuscript.

Field-specific reporting

Please select the one below that is the best fit for your research. If you are not sure, read the appropriate sections before making your selection.

☐ Life sciences ☒ Behavioural & social sciences ☐ Ecological, evolutionary & environmental sciences

For a reference copy of the document with all sections, see nature.com/documents/nr-reporting-summary-flat.pdf

Behavioural & social sciences study design

All studies must disclose on these points even when the disclosure is negative.

Study description

This manuscript reports a quantitative longitudinal study conducted between 1992 and 2020.

Research sample

This manuscript is based on primary survey data from the Young in Norway Study that includes a population-based sample of Norwegians (N = 2,215). The rationale for the chosen study sample was to secure a random cohort of adolescents for longitudinal observation throughout their life course. At T1 (1992), participants were junior and senior high school students in grades 7-12. Participant selection was based on randomized stratified sampling. Each grade was equally represented. The T1 sample was equally distributed according to gender and age (12 to 20 years). In the T5 sample (used in this study), 57.4% of participants were women, and 42.6% were men (as compared to 53% of men and 47% of women in the Norwegian population). Most participants (93.6%) were ethnic Norwegians; 6.4% of participants had some immigrant background (i.e., were born abroad or had at least one parent who was born abroad) as compared with about 17% in the overall population. This under-representation of immigrants might have been due to lower percentages in the population at the initial data collection.

Sampling strategy

The collection of the data used in this study started in 1992 (T1) and aimed at a national representative sample. Here, we used the existing sample followed for 28 years and therefore did not employ any statistical methods to predetermine sample size. Power analyses showed that even small effects sizes of r = .07 can detected with a power exceeding 90% with a sample size of N = 2,215.

To ensure representativeness, initial data collection covered 67 schools across Norway, grades 7-12, selected from stratified areas (5 strata). Each grade was equally represented, and cluster-sampling was applied with the school as the unit.

Data collection

Data were collected in a paper and pencil format (T1 - T4) and with the Nettskjema data collection tool (T5), a tool approved at the University of Oslo for collecting sensitive data. More information: https://www.ulo.no/english/services/it/adm-services/nettskjema/about-nettskjema.html

	In addition to measures used in this study (see Supplementary Information, Table S1) participants responded to a number of other measures. No experiments were conducted as part of this study, thus random assignment and experimenter blinding are not applicable.
Timing	Data collection took place at five time points: T1 (May to November 1992), T2 (January to June 1994), T3 (January 1999 to June 2000, with a majority of 93% of the sample data collected in 1999), T4 (June 2005 to June 2006, with a majority of 83% of the sample data collected in 2005), and T5 (September 2020 to May 2021, with a majority of 60% of the sample data collected in 2020).
Data exclusions	Because the outcome measure of interest (i.e., conspiracy mentality) was included at T5, only data from participants who had completed this wave were used in the current study.
Non-participation	The initial sample (T1) consisted of 11,985 participants, T2 (1994; N = 3,507), T3 (1999; N = 2,924), T4 (2005; N = 2,890), and T5 (2020; N = 2,215). The greatest drop was observed between T1 and T2 for the following reasons: Three schools included at T1 (1992) for participation in the first wave only, and were not part of the follow-up (T2). At one other school project ID records were lost due to force majeure. Approximately half of the remaining 9,679 students from 63 schools eligible to complete the T2 questionnaire, had completed the 3-year track at their junior or senior high school at T2 and therefore left their original school. Among those who were still at their original schools, 91.8 % responded. These participants were followed up at T3 using postal data collection, with the response rate of 83.8%, and at T4, with the response rate of 82.4%. At T5, 14 participants had passed away, and 5 withdrew their consent. In all, 2,214 of 2,587 eligible participants completed the questionnaire, setting the response rate to 85.6 %.
Randomization	This study does not involve experimental groups, thus randomization was used only at the initial data collection to obtain a representative sample. We recruited junior and senior high school students, from 67 schools in grades 7-12 selected in a two-stage manner from stratified areas. Each grade was equally represented. Cluster-sampling was applied with the school as the unit. Every school in Norway was included in the register from which the schools were selected. The schools in Norway were divided into 5 geographical strata, ensuring the representativeness on the urban-rural dimension.

Reporting for specific materials, systems and methods

We require information from authors about some types of materials, experimental systems and methods used in many studies. Here, indicate whether each material, system or method listed is relevant to your study. If you are not sure if a list item applies to your research, read the appropriate section before selecting a response.

Materials & experimental systems

n/a	Involved in the study
☒	☐ Antibodies
☒	☐ Eukaryotic cell lines
☒	☐ Palaeontology and archaeology
☒	☐ Animals and other organisms
☒	☐ Clinical data
☒	☐ Dual use research of concern
☒	☐ Plants

Methods

n/a	Involved in the study
☒	☐ ChIP-seq
☒	☐ Flow cytometry
☒	☐ MRI-based neuroimaging

Plants

Seed stocks	*Report on the source of all seed stocks or other plant material used. If applicable, state the seed stock centre and catalogue number. If plant specimens were collected from the field, describe the collection location, date and sampling procedures.*
Novel plant genotypes	*Describe the methods by which all novel plant genotypes were produced. This includes those generated by transgenic approaches, gene editing, chemical/radiation-based mutagenesis and hybridization. For transgenic lines, describe the transformation method, the number of independent lines analyzed and the generation upon which experiments were performed. For gene-edited lines, describe the editor used, the endogenous sequence targeted for editing, the targeting guide RNA sequence (if applicable) and how the editor was applied.*
Authentication	*Describe any authentication procedures for each seed stock used or novel genotype generated. Describe any experiments used to assess the effect of a mutation and, where applicable, how potential secondary effects (e.g. second site T-DNA insertions, mosiacism, off-target gene editing) were examined.*
